# Involving Parents in Cognitive Behavioral Therapy for Children and Adolescents with Conduct Problems: Goals, Outcome Expectations, and Normative Beliefs About Aggression are Targeted in Sessions with Parents and Their Child

**DOI:** 10.1007/s10567-024-00486-3

**Published:** 2024-06-08

**Authors:** Walter Matthys, Dennis J. L. G. Schutter

**Affiliations:** 1 Department of Clinical Child and Family Studies, Utrecht University, Heidelberglaan 1, 3584 CS Utrecht, The Netherlands; 2 Department of Experimental Psychology, Helmholtz Institute, Utrecht University, Heidelberglaan 1, 3584 CS Utrecht, The Netherlands

**Keywords:** Conduct problems, Cognitive behavioral therapy, Goals, Outcome expectations, Normative beliefs, Schemas

## Abstract

Children and adolescents with conduct problems participate in Cognitive Behavioral Therapy (CBT), either in individual or group format, in view of learning social problem-solving skills that enable them to behave in more independent and situation-appropriate ways. Parents must support their child’s learning processes in everyday life and therefore these processes need attention in CBT sessions in which parents and their child participate. The social problem-solving model of CBT previously described (Matthys & Schutter, Clin Child Fam Psychol Rev 25:552–572, 2022; Matthys & Schutter, Clin Child Fam Psychol Rev 26:401–415, 2023) consists of nine psychological skills. In this narrative review we propose that instead of addressing each skill separately in sessions with both parents and their child, therapists work on three schemas (latent mental structures): (1) goals, (2) outcome expectations, and (3) normative beliefs about aggression. Based on social-cognitive and cognitive neuroscience studies we argue that these three schemas affect five core social problem-solving skills: (1) interpretation, (2) clarification of goals, (3) generations of solutions, (4) evaluation of solutions, and (5) decision-making. In view of tailoring CBT to the individual child’s characteristic schemas and associated social problem-solving skills, we suggest that children and adolescents participate in individual sessions with their parents. The therapist uses Socratic questioning in order to find out characteristic schemas of the child, encourage reflection on these schemas, and explore alternative schemas that had previously been outside the child’s attention. The therapist functions as a model for parents to ask their child questions about the relevant schemas with a view of achieving changes in the schemas.

Children and adolescents with conduct problems in the clinical range either meet criteria of oppositional defiant disorder (ODD) or conduct disorder (CD) according to the *Diagnostic and Statistical Manual of Mental Disorders* (fifth edition, *DSM-5*, American Psychiatric Association, 2013) or show symptoms in the clinical range of defiant behavior, irritability, aggressive behavior, or antisocial behavior on a standardized measure of psychopathology (e.g., the Achenbach System of Empirically Based Assessment (ASEBA); Achenbach, 2009). Effective psychological treatment of conduct problems are needed given the range of negative outcomes in adulthood (Fergusson et al., 2005; Kim-Cohen et al., 2003) as well as high costs in terms of service utilization across all three domains of criminal justice, health, and social welfare (Rivenbark et al., 2018).

Parent training programs are among the well-studied psychological interventions for the prevention and treatment of conduct problems in children and adolescents. According to the most recent meta-analysis of parent training including 241 studies the overall mean effect size at post-intervention (up to 3 months after the termination of the program) was positive for antisocial behavior (*d* = 0.47) (Beelman et al., 2023). However, the effect size for antisocial behavior decreased in short-term follow-ups (3–12 months) to *d* = 0.22 and long-term follow-ups (12 months or more) to *d* = 0.12. Effect sizes depended on the risk level of the target group in antisocial behavior (universal prevention, selective prevention, indicated prevention, treatment) and were marked at follow-up. Whereas universal and selective prevention had very low effects in the 12 months or more follow-up (*d* = 0.05 for universal and *d* = 0.07 for selective prevention), effect sizes for indicated prevention (*d* = 0.29), and treatment (*d* = 0.22) were small.

One way to increase effectiveness and achieve sustainable effects of psychological interventions for the treatment of conduct problems in children aged from 7 years on is to combine Parent Training with Cognitive Behavior Therapy (CBT). CBT provides children and adolescents with social problem-solving skills that enable them to behave in more independent and situation-appropriate ways (Matthys & Schutter, 2022). Examples of programs are Problem-Solving Skills Training combined with Parent Management Training (Kazdin et al., 1992) and the Coping Power program including a child component (Lochman et al., 2008) and a parent component (Wells et al., 2008). These programs have proven successful in clinical samples (Helander et al., 2018; Kazdin et al., 1992; Van de Wiel et al., 2007; Zonnevylle-Bender et al., 2007). Likewise, there is empirical evidence for effectiveness of Aggression Replacement Training, a CBT program for delinquent adolescents (Hornsveld et al., 2015).

In the meta-analysis by McCart et al. (2006), the effect size of CBT in children and adolescents with conduct problems is *d* = 0.35. This meta-analysis found a positive relationship between age and effect size, showing that as youth age and progress into more advanced levels of cognitive development, they benefit more from CBT. CBT programs may need modification as most programs were developed during the last three decades of the previous century and have been only slightly updated throughout the years. CBT for children and adolescents with conduct problems can be based on the original model of Problem Solving Therapy developed by D’Zurilla and Goldfried (1971), subsequent elaborations of this model (D’Zurilla, 1988), relevant meta-analyses (Bell & D’Zurilla, 2009; Malouff et al., 2007), and the related social information-processing model described by Dodge in 1986 and later adapted by Crick and Dodge (1994) (for a discussion of these models, see Matthys & Schutter, 2022). We consider the psychological functions in the social information-processing model developed by Crick and Dodge (1994) similar to social problem-solving skills (Matthys et al., 1999). In view of an update of the social problem-solving model of CBT, empirical studies on psychological functions involved in social problem-solving were considered (Matthys & Schutter, 2022), as well as moral thinking and empathy related to social problem-solving skills (Matthys & Schutter, 2023). These two reviews resulted in a model of social problem-solving that consisted of nine psychological (social problem-solving) skills: (1) recognition of problematic social situations, (2) recognition of facial expressions in view of initiating social problem-solving, (3) emotional awareness and regulation (4) behavioral inhibition and working memory, (5) interpretation of social problems, (6) clarification of goals, (7) generation of solutions, (8) evaluation of solutions, and (9) decision-making (Matthys & Schutter, 2023) (see Table 1).

**Table 1:** What children and adolescents learn in Cognitive Behavioral Therapy

	Psychological skill
1	*Recognition of problematic social situations*
	Which social situations are problematic for me? This is important to know in view of starting thinking in a situation that might be difficult for me to handle
2	*Recognition of facial expressions*
	What do other person’s facial expressions tell me about their feelings and about a possible social problem? If the other person feels anxious, sad, or angry this person can expect me to respond to this. I really need to think now
3	*Emotional awareness and regulation*
	What do I feel myself? And in case my own feeling (e.g., anxiety, sadness, anger) is too strong, what can I do to cope with this feeling?
4	*Behavioral inhibition and working memory*
	I shouldn’t act right away. Rather, I should think first and concentrate on what is the problem and how to solve it
5	*Interpretation*
	When I see that someone else has done something bad to me, I shouldn’t always think this person did that on purpose. Maybe I’m inclined to think this because I’m convinced that’s the way how people treat each other. On the other hand, when I see that the other person feels bad I should try to understand what did happen. Maybe I don’t see that I did hurt the other because I’m not used to paying attention to it. And what do I feel myself when I see how this person feels?
6	*Clarification of goals*
	When I want to solve this problem, what is my goal? If I know my goal, I can better think about what to do. Do I want to get my way or revenge for wrongs done? Or do I want to work things out and find a solution together with this person? And if this person feels bad, am I willing to help that person?
7	*Generation of solutions*
	In a difficult situation I must try to come up with one or more solutions. Maybe I think that only aggressive solutions work. Perhaps there are also solutions that bring both me and the other person benefits? To find such ‘kind’ solutions, I need to think what the problem actually is (step 5) and what I want to achieve (step 6)
8	*Evaluation of solutions*
	Then, I would do well to think about consequences of solutions for me, the other person, and our relationship, both on the short and long-term. I may have difficulty believing that positive consequences can also be expected to result from solutions that are clearly not aggressive but ‘kind’ (or constructive) instead. Related to this, I would do well to consider if the solutions I’m thinking about do not harm the other person
9	*Decision-making*
	In the end I choose the best solution for both of us. For this, I have to make connections between previous steps: What is the problem? What is my goal? What are possible solutions? And what is the solution that is most beneficial for the other person and myself? This can be difficult for me. But many positive experiences with appropriate solutions will help me making correct decisions

The learning processes involved in changing social problem-solving abilities are assumed to be slow. Working on these psychological skills only during CBT sessions is not sufficient and needs support in everyday life (“in vivo practice”; Kazdin et al., 1989). Parents, therefore, must be involved in the delivery of CBT of their child in view of supporting the child’s learning processes. Rather than working on each of nine psychological functions mentioned above in therapy sessions (Matthys & Schutter, 2023), we propose parents and children or adolescents attend to three schemas which simplify the cognitive tasks involved in social problem-solving: (1) goals, (2) outcome expectations, and (3) normative beliefs about aggression.

Focusing on a limited number of schemas is in line with research in cognitive science demonstrating that individuals when confronted with the overwhelming amount of stimulus information that is present in most situations, often rely on schemas to simplify the cognitive tasks involved in processing that information (Crick & Dodge, 1994; Fiske & Taylor, 1991). In their model, Crick and Dodge (1994) propose that a mental representation of past events is stored in long-term memory. This memory is integrated with other memories into a general mental structure or schema that guides the processing of future social cues. In particular, a schema may be activated in current social situations by situational cues. Thus, a schema may affect situation-specific information processing or social problem-solving. Schemas, therefore, facilitate social information-processing (de Castro & van Dijk, 2018) or social problem-solving.

The aim of the present narrative review is, first, to examine how the three schemas (i.e., goals, outcome expectations, and normative beliefs about aggression) affect five core social problem-solving skills (i.e., interpretation, clarification of goals, generation of solutions, evaluation of solutions, decision-making) in children and adolescents with conduct problems. To this end, we review social-cognitive and cognitive neuroscience research on goals, outcome expectations and normative beliefs about aggression in children and adolescents with conduct problems in view of their role in social problem-solving. Second, we discuss how goals, outcome expectations and normative beliefs about aggression can become part of CBT sessions with these children or adolescents and their parents (Fig. 1).

**Fig. 1:**
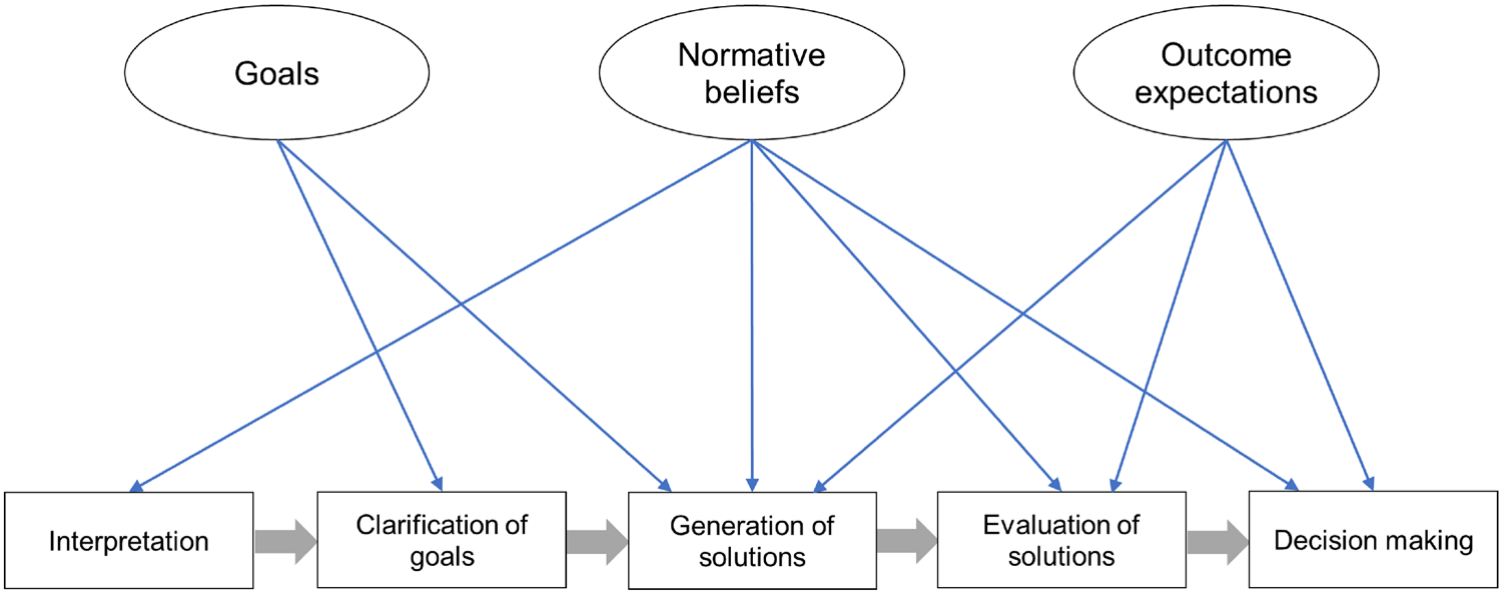
Schemas and social problem-solving skills

In the current review, the term children refers to both children and adolescents. The term adolescents is only used in studies of adolescents only, and when making statements about adolescence specifically. Furthermore, in social-cognitive studies conduct problems in children are not often clinically defined (e.g., fulfilling criteria of conduct disorder). Rather, conduct problems are defined in terms of scores on rating scales for aggression (e.g., fighting, threatening others) and/or antisocial behavior (e.g., stealing, destroying others’ property). Finally, some studies examine children who exhibit characteristics that qualify for the “with limited prosocial emotions” specifier (previously called callous-unemotional or psychopathic traits), which include lack of empathy, lack of remorse or guilt, shallow or deficient affect, and unconcerned about one’s own performance (American Psychiatric Association, 2013).

## Goals: A Social-Cognitive Perspective

Goals can be defined as internal representations of desired states, where states are broadly defined as outcomes, events or processes (Austin & Vancouver, 1996). A distinction needs to be made between clarification of goals as a social information-processing step or social problem-solving skill on one hand (Table 1) and goals as orientations, tendencies or schemas toward producing desired outcomes on the other hand (Crick & Dodge, 1994; Erdley & Asher, 1999). As specified by Crick and Dodge (1994), children bring goal orientations or tendencies to a social problem situation (i.e., goals as schemas), but also revise those goals in response to immediate social stimuli (i.e., clarification of goals as a social problem-solving step). In other words, goals are defined as motivational orientations stored in long-term memory and based on past social experiences (i.e., goals as schemas). These goals are shaped in response to a specific social situation (i.e., clarification of goals as a social problem-solving step).

Several studies have shown deviant goals in children with conduct problems. In these studies goals are considered a social problem-solving step and assessed using vignettes of social problems (i.e., hypothetical peer situations). For example, adolescents incarcerated for having committed one or more violent criminal acts and adolescents rated by teachers as high-aggressive were more likely to choose a hostile goal when solving social problems than adolescents rated by their teachers as low-aggressive (Slaby & Guerra, 1988). In another study goals were assessed in children who varied in their behavioral responses (i.e., aggression vs. withdrawal vs. problem solving) to ambiguous provocations. Fourth and fifth grade children who reacted aggressively to hypothetical situations involving ambiguous provocation, endorsed more hostile goals compared to withdrawn and problem-solving responders. Specifically, the aggressive responders were more focused on punishing the protagonist and defending one self, and were less concerned with preserving a relationship with the protagonist or dealing with the provocation in a constructive manner (Erdley & Asher, 1996). Also, among adolescent boys consistent associations have been found between delinquent, substance-using, and behavioral difficulties on the one hand and high goal values for dominance and revenge, and low goal values for affiliation on the other hand. Dominance proved to be the most sensitive correlate of these negative outcomes (Lochman et al., 1993). In another study, 9–12 year old proactive-aggressive children were less likely than their peers to select relationship-enhancing goals during social interaction. Rather, they were more likely to prefer goals that are instrumental in nature (Crick & Dodge, 1996).

Among the sources of goal orientations Crick and Dodge (1994) mention the role of feelings. For example, feeling angry might serve as an impetus for a retaliatory goal. In addition, the intensity with which children experience emotions and their emotion regulation capacities are relevant to mention here. Children who are overwhelmed by their own emotions may choose hostile goals to reduce arousal (Lemerise & Arsenio, 2000). In a longitudinal study of fourth through seventh graders, aggressive children who found it difficult to regulate their angry and anxious emotions and inhibit their behaviors, endorsed revenge goals at initially higher levels and continued to endorse revenge at higher levels for at least two more years (McDonald & Lochman, 2012).

Goals have also been studied in delinquent adolescents with callous-unemotional traits. Both callous-unemotional traits and prior violence were significantly correlated with a greater endorsement of goals related to dominance, revenge, forced respect, and overall conflict escalation, as well as lower endorsement of goals related to conflict avoidance and relationship building (Pardini, 2011).

A meta-analytic study examined associations between goals and aggression in children (Samson et al., 2012). Mean positive correlation of 0.15 between antisocial goals and aggression and an inverse correlation of 0.14 between prosocial goals and aggression were comparable to mean correlations between aggression and hostile attribution (*r* = 0.17) (de Castro et al., 2002). These findings suggest that increased likelihood of aggression is associated with a low propensity to endorse prosocial goals and a high propensity to endorse antisocial goals for social interaction. The authors conclude that goals for social interaction should be targeted as a potential avenue to reduce aggression. Likewise, a meta-analysis examined associations between goals and bullying (Samson et al., 2022). Children who displayed more bullying behavior were more likely to endorse status/power goals (*r* = 0.16) and antisocial goals (*r* = 0.27), and to disregard prosocial goals (*r* = −0.10).

In contrast to the studies discussed above in which situation-specific goals were investigated using vignettes depicting hypothetical social problem situations, Ojanen and colleagues (2005) developed a procedure to assess social goals as schemas, that is the Interpersonal Goals Inventory for Children. Sample items for each subscale are as follows: agentic (“others think you are smart”); agentic and communal (“you are able to tell others how you feel”); communal (“real friendship develops between you and others”); submissive and communal (“others accept you”); submissive (“you are able to please others”); submissive and separate (“your peers do not laugh at you”); separate (“you keep others at a suitable distance”); and agentic and separate (“the group does what you say”).

A longitudinal study by Ojanen and Findley-van Nostrand (2014) using this procedure examined agentic goals reflecting agency or power, and communal goals reflecting closeness in adolescents aged 12–14 years. Agentic goals predicted increased relational aggression and communal goals predicted decreased physical aggression. The authors suggest to target goals to reduce aggression and refer to an intervention study that was successful in changing goals. Indeed, in a study of Second Step, a universal prevention program including problem-solving, intervention children (fifth and sixth grade) were more likely to prefer prosocial goals than control children; intervention children also behaved less aggressively than control children (Frey et al., 2005). These authors conclude that in order to act in socially responsible ways, children must possess both the relevant skills and the motivation to use those skills.

The procedure to assess social goals as schemas or trait-like motivational orientations developed by Ojanen and colleagues (2005) was also used in a study among nonclinical adolescents with psychopathic traits (affective–interpersonal and antisocial–impulsive) (Ojanen & Findley-van Nostrand, 2019). Only the affective–interpersonal dimension of psychopathy was uniquely positively linked to agentic goals and negatively to communal goals. In addition, only the affective–interpersonal dimension was indirectly related to proactive aggression via agentic goals. Finally, the procedure to assess goals as schemas developed by Ojanen and colleagues (2012) was used in a study of the role of narcissism in aggression among adolescents. Narcissism was associated with physical aggression via dominance goals for boys and with relational aggression via dominance goals for both boys and girls.

In summary, a distinction needs to be made between goals as schemas and the clarification (or identification) of one or more goals as one of the nine social problem-solving steps. In line with Crick and Dodge (1994) we assume that there is an association between both. Findings from a study in typically developing children indicate that children’s social goals have trait-like, as well as situation-specific characteristics (Ojanen et al., 2007). Social-cognitive research suggests that aggressive and delinquent children’s goals are characterized as hostile, punishing, revengeful, dominant, conflict escalating, and focused on status and power. In contrast, when compared to well-adjusted children the goals they construct are less affiliative, relationship building and enhancing, conflict avoidant and aimed at closeness. Finally, for a neuroscience perspective on goals, see section “Outcome Expectations: A Cognitive Neuroscience Perspective on Aggression Motivation.”

## Outcome Expectations: A Social-Cognitive Perspective

According to Crick and Dodge (1994), after the generation of the responses, the responses are evaluated based on outcome expectations, that is the children’s ideas about what is likely to occur in a social interaction after the enactment of a behavioral response. Children with aggressive behavior expect aggressive behavior to lead to favorable outcomes for they have learned that aggression reduces aversive treatment by other people (see principle of negative reinforcement and Patterson’s Coercive Theory, 1982). For example, aggressive children are more confident that aggression will produce tangible rewards and reduce aversive treatment by others compared to non-aggressive children (Perry et al., 1986). Similarly, adolescents incarcerated in a state juvenile correctional facility for having committed one or more violent criminal acts when compared to aggressive and non-aggressive high-school students were more likely to hold a set of beliefs supporting the use of aggression. Those beliefs included the view that aggression is a legitimate response, increases self-esteem, helps to avoid a negative image and does not lead to suffering by the victim (Slaby & Guerra, 1988).

A study with incarcerated adolescent boys aged 13–18 years investigated whether the relation between aggressive outcome expectations was specific to the proactive subtype of aggression as opposed to the reactive subtype (Smithmyer et al., 2000). Proactive aggression is instrumental and occurs without provocation, whereas reactive aggression occurs in response to a perceived provocation or threat. Proactive aggression was positively related to the tendency to expect positive outcomes for aggressive acts. No such conclusion was supported for reactive aggression. This finding is consistent with the view that proactive aggression is goal oriented in nature (Smithmyer et al., 2000); see also next section, “Outcome Expectations: A Cognitive Neuroscience Perspective on Aggression Motivation.”

There is also evidence that atypical outcome expectations are related to callous-unemotional traits. In a study with adjudicated youth, higher callous-unemotional traits were related to increased expectations about the positive consequences of aggression (i.e., tangible rewards, dominance) and decreased expectations about the negative consequences of deviant behavior (i.e., punishment) (Pardini et al., 2003). In the study by Pardini (2011) previously discussed with regard to goals, expectancies and values regarding victim suffering following aggression were investigated in delinquent adolescents with callous-unemotional traits. This is interesting as goals function as orientations toward producing desirable outcomes. While neither callous-unemotional traits nor prior violence was significantly associated with expectations that aggressive acts would cause victim suffering, both were significantly correlated with decreased concern about the victim suffering as the result of aggression. The author concludes that adolescents with callous-unemotional traits seem to be aware that their aggressive behavior will cause others to suffer, but they do not care when it does.

Elowsky and colleagues (2022) examined the outcome expectations and values regarding the consequences of aggression in adolescents with conduct disorder, and the role of callous-unemotional traits and irritability. Callous-unemotional traits were associated with decreased expectation that aggression would result in feelings of remorse and victim suffering, as well as decreased concern that aggressive acts would result in punishment and victim suffering. Irritability was associated with increased expectations and concern that aggression would indeed result in dominance and forced respect. This study suggests that callous-unemotional traits and irritability are associated with different forms of maladaptive outcome expectations and values regarding the consequences of aggression.

In summary, social-cognitive research suggests that children with conduct problems or aggressive behavior expect that aggression will produce tangible rewards, will reduce aversive treatment by others, is a legitimate response, increases self-esteem and helps avoid a negative image. Callous-unemotional traits are associated with decreased concern that aggressive acts will result in victim suffering.

## Outcome Expectations: A Cognitive Neuroscience Perspective on Aggression Motivation

As previously discussed, according Crick and Dodge’s social-cognitive model (1994) goals function as orientations toward producing particular outcomes. Similarly, according to cognitive neuroscience goals are associated with outcomes, at least in instrumental aggression (Blair, 2022). Indeed, instrumental aggression is a behavior motivated by the desire to achieve a particular goal (Blair, 2022). By contrast, reactive aggression is unplanned and impulsive; the aggression is not initiated to achieve a goal and is often accompanied by negative affect (fear, anger, sadness, frustration, and irritation) (Blair, 2022). However, the distinction between reactive aggression and instrumental or proactive aggression is sometimes not straightforward (Kempes et al., 2005). Behavior that looks like an instance of reactive aggression (e.g., a child being angry because he is denied the toy) can have a proactive goal (e.g., being angry will help him to obtain the toy; Kempes et al., 2005). On the other hand, a seemingly proactive aggressive act can be a delayed reaction to an earlier event (e.g., a boy takes his revenge after being teased a couple of hours before) (Kempes et al., 2005). Blair (2022) also recognizes that the dichotomous view of reactive and instrumental aggression is too rigid. Here, we adopt Blair’s (2022) cognitive neuroscience model of aggression motivation, in particular of instrumental/proactive aggression and reactive aggression in so far as the latter also has an instrumental/proactive component (Blair, 2022). In his theory, Blair focuses more on outcomes and less on goal orientations.

Blair (2022) conceptualizes motivation of aggression in terms of the neuro-cognitive systems involved in aggression. For appropriate aggression motivation, the individual must: first, *represent the value of the consequences of the action* (reward to the self and consequences for the victim); second, *be responsive to the consequences to the victim* (the victim’s distress); third, be able to *stop or alter the aggressive behavior* (response control and inhibition) if the aversive consequences become too great.

With regard to *representing the value of the consequences of the action*, an individual engaging in instrumental aggression has a motive (Blair, 2022). The individual uses aggression (e.g., mugging someone) to gain an outcome (e.g., the victim’s money; Blair, 2022). The outcome the individual wishes to achieve is rewarding, even if the aggressor’s goal is to avoid an aversive outcome (Blair, 2022). Reinforcement based decision-making studies show that reduced neural responsiveness to reward puts an individual at risk of poor decision-making because response choices are less guided by expectations that an action will result in reward relative to punishment (Blair et al., 2018). Children with conduct problems have been found to show reduced activation in the orbitofrontal cortex, ventromedial prefrontal cortex, anterior cingulate cortex and striatum during anticipation of rewards (Finger et al., 2011; Hawes et al., 2021; White et al., 2013). In addition, while performing a decision-making task adolescents with conduct problems showed significantly reduced use of expected value information about the consequences of a choice in the bilateral caudate and posterior cingulate cortex during the avoidance of suboptimal responses (White et al., 2014). Also, while performing a passive avoidance task conduct problem adolescents’ reduced representation of expected values when making avoidance responses within bilateral anterior insula cortex, inferior frontal gyrus and striatum was associated with greater levels of conduct problems (White et al., 2016). In sum, problems in representing the value of the consequences of an action can impede children’s ability to make appropriate decisions when solving social problems; as a result, the aggressive and antisocial behavior displayed by children and adolescents with conduct problems may be less instrumental, planned or proactive than it may first seem. This is in line with the study by Smeets and colleagues (2017) in a large clinical sample which showed that no proactive-only group could be determined.

Second, the individual must be *responsive to the consequences for the victim*; that is, their distress (Blair, 2022). A meta-analysis showed a strong association between antisocial behavior and deficits in recognizing fearful expressions (Marsh & Blair, 2008). Consistent with this, in a functional magnetic resonance imaging (fMRI) study children and adolescents with conduct problems and callous-unemotional traits showed reduced amygdala responsiveness during the presentation of fearful facial expressions in comparison to healthy controls and youth with Attention-Deficit/Hyperactivity Disorder (ADHD) (Marsh et al., 2008). Interestingly, functional connectivity analyses demonstrated lower correlations between the amygdala and ventromedial prefrontal cortex in youth with conduct problems and callous-unemotional traits as compared to healthy controls and youth with ADHD (Marsh et al., 2008). Impairments in amygdala-ventromedial prefrontal cortex connectivity are suggested to be associated with antisocial behavior as a result of instrumental behavior that is inappropriately modulated by others’ distress (Marsh et al., 2008). In addition, adolescents with conduct problems and psychopathic traits showed reduced activity in the rostral anterior cingulate cortex, ventral striatum, and amygdala in response to observing increased pain in others (Marsh et al., 2013). Also, reduced activity in the insula while viewing others being harmed was related to children’s greater number of conduct disorder symptoms and callous-unemotional traits (Michalska et al., 2016). In sum, the reduced response to the distress of others is associated with reduced learning to avoid actions that harm other individuals (the individual finds the “punishment” of the other individual’s distress less aversive), increasing the risk that aggression might be used (Blair, 2003).

Third, individuals must be *able to stop or alter their behavior* if the aversive consequences for the other person become too great (Blair, 2022). Response control or inhibition refers to the control of actions that interfere with goal-driven behavior (Blair, 2022). In their review of fMRI studies Blair and colleagues (2018) conclude that studies examining different paradigms involving response inhibition report no differences in recruitment of brain regions implicated in response control (i.e., inferior frontal gyrus, anterior insula cortex, dorsomedial frontal cortex) between children with conduct problems and controls. The authors note that several of these studies excluded youth with conduct problems with comorbid ADHD. A study that did not control for the presence of ADHD showed reduced anterior insula activity on a cognitive interference (Stroop) task. The extent of impairment did not particularly relate to severity of conduct problems, but did positively correlate to ADHD symptom severity (Hwang et al., 2016). As ADHD often is associated with conduct problems (Angold et al., 1999), impaired response inhibition may be a characteristic of children and adolescents with conduct problems. In sum, disruption in response control or inhibition likely increases the risk of aggression.

In the three neurocognitive systems involved in aggression dopaminergic and serotonergic functioning can be considered (Blair, 2022). Decreased dopaminergic functioning has been shown in children with conduct problems (Matthys et al., 2013). Blair (2022) suggests that within the model presented above decreased dopaminergic functioning might be expected to increase aggression by: first, compromised reinforcement-based decision-making (i.e., there is considerable dopaminergic innervation of systems involved in reinforcement-based decision-making); second, compromised distress cue processing (i.e., dopamine depletion disrupts distress expression processing); third, compromised response control (i.e., dopamine depletion disrupts response inhibition). There is also evidence for decreased serotonergic functioning in children with conduct problems (Matthys et al., 2013). This is relevant as serotonin may be important for appropriate representation of expected value in decision-making (Blair, 2022).

In summary, outcome expectations are considered here in terms of Blair’s (2022) motivational theory of aggression consisting of: (1) the representation of the values of the consequences of an action; (2) the responsiveness to the consequences to the victim; (3) the ability to stop or alter (aggressive) behavior. In all three neurocognitive functions deviances have been found in fMRI studies in children with conduct problems. As a result of difficulties in the representation of the value (rewarding/positive, punishing/negative) of the consequences of actions, aggressive and antisocial behavior may be in part unplanned. In addition, decreased responsiveness to the consequences to the victim and difficulties in stopping or altering behavior can increase the damage children cause the victims and ultimately themselves.

## Normative Beliefs About Aggression: A Social-Cognitive Perspective

Slaby and Guerra (1988) studied beliefs supporting aggression in violent juvenile offenders, high-aggressive high school students, and low-aggressive high school students. Violent juvenile offenders when compared to low-aggressive high school students were more likely to hold a set of beliefs supporting the use of aggression. These beliefs include opinions that aggression is a legitimate response, increases self-esteem, helps avoid a negative image, and does not lead to suffering by the victim (Slaby & Guerra, 1988).

In agreement, Huesmann and Guerra (1997) introduced the concept normative beliefs, defined as the individual’s cognitive standards about the acceptability or unacceptability of a behavior; children acquire normative beliefs through observation, experience, and tuition they receive from peers, parents, and teachers. Huesmann and Guerra (1997) found that in fourth and fifth graders individual differences in normative beliefs that aggressive forms of behavior are socially acceptable and appropriate, predict an increase in aggressive behavior as sixth graders. Huesmann and Guerra (1997) hypothesized three ways in which normative beliefs affect children’s aggressive behavior. First, normative beliefs may affect the way in which children perceive or interpret the behaviors of others; the more children approve of aggression, the more likely they may be to perceive hostility in others, even if no hostility is present. Second, normative beliefs in support of aggression may cue the retrieval of aggressive scripts for social behavior. In other words, normative beliefs may help generating aggressive solutions to social problems. Finally, if normative beliefs act as filters to eliminate “inappropriate” behaviors from children’s repertoires, children with normative beliefs in support of aggression are less likely to reject aggressive solutions once they have thought of them as solutions to social problems. Thus, normative beliefs may play a role in the evaluation step of social problem-solving.

The hypotheses by Huesmann and Guerra were confirmed in a study by Zelli and colleagues (1999). Individual differences in retaliation approval among third graders predicted individual differences in fifth graders’ aggressive behavior; nearly 50% of this effect could be attributed to three social information-processing steps: (1) attribution of hostile intentions, (2) generation of aggressive responses, and (3) positive evaluation of aggressive responses (Zelli et al., 1999).

In addition, positive evaluation of aggressive behavior, including social acceptability and moral appropriateness of aggression, incremented the prediction from externalizing behavior in early adolescence to later antisocial problems (Fontaine et al., 2002). The distinction between reactive and proactive aggression may be relevant here. Higher levels of proactive aggression in adolescents were associated with lower moral concerns (i.e., deny or minimize negative consequences for others) regarding one’s aggression (Arsenio et al., 2009). There is also evidence that normative beliefs are related to callous-unemotional traits. In a study with adjudicated youth, higher callous-unemotional traits were related to increased expectations and values associated with the positive consequences of aggression (i.e., tangible rewards, dominance) and decreased expectations and values associated with the negative consequences of deviant behavior (i.e., punishment) (Pardini et al., 2003).

A longitudinal study in adolescents investigated whether the justification of violence schema predicted social information-processing, and social information-processing in turn predicted aggressive behavior (Calvete & Orue, 2012). The justification of violence schema predicted access to forms of aggressive behavior when the adolescent imagined him- or herself in an ambiguous social encounter (i.e., response generation), and these thoughts predicted reactive aggressive behavior. The authors concluded that interventions should incorporate the modification of dysfunctional schemas. In another longitudinal study the relationship between justification of violence and child-to-parent aggression was assessed and it was tested whether social information-processing mediates this association (Orue et al., 2021). The justification of violence predicted hostile attribution, aggressive response access, and the anticipation of positive consequences. Furthermore, the justification of violence predicted child-to-parent aggression both directly and through aggressive response access.

Bellmore and colleagues (2005) found that adolescents who believed in the appropriateness of aggression selected hostile response options that resulted in subsequent physical, verbal, and indirect bullying behavior. The influence of normative beliefs on adolescents’ aggressive reputations among their teachers and classmates, was shown to be indirect, that is adolescents’ response selections mediated the influence that their beliefs had on their aggressive reputations. In view of interventions, the authors commented that short-term effects might be obtained by simply changing response selections, but that longer-lasting effects almost certainly depend on making changes in the schemas that influence social information-processing skills.

Finally, community violence exposure has been associated with subsequent aggressive behavior; normative beliefs supporting aggression are one potential mechanism underlying this relation (Pittman, 2023). According to a systematic review, findings across studies generally supported the notion that normative beliefs about aggression mediate relations between community violence exposure and aggressive behavior (Pittman, 2023).

In summary, social-cognitive research suggests that normative beliefs about aggression affect aggressive behavior through hostile interpretation, generation of aggressive solutions, positive evaluation of aggressive solutions, and selection of aggressive solutions.

## Normative Beliefs About Aggression: A Cognitive Neuroscience Perspective on Moral Thinking

Aggressive and antisocial behaviors can be considered moral transgressions. Indeed, harming others physically or psychologically as well as stealing other’s properties belong to the moral domain. In the context of normative beliefs, care-based norms are specifically relevant. Care-based norms are norms regarding actions that might harm others (Blair, 2023). Care-based morality refers to those forms of moral reasoning that concern actions that harm others (Blair, 2007). Cognitive neuroscience studies show the role of the sensitivity to interpersonal harm in moral cognition (Decety & Cowell, 2018).

Stimulus-reinforcement learning allows the individual to associate a valence with a stimulus, that is to learn whether something is good or bad (Blair, 2017). In particular, Blair (2017) argues that one’s sense of “badness” of care-based moral transgressions is associated with an aversive unconditioned stimulus, that is the distress of the other individual. Impairments in stimulus-reinforcement learning and in responsiveness to the distress of other individuals disrupt individual’s ability to learn the emotion-based sense of badness of care-based moral transgressions (Blair, 2017).

The amygdala plays an essential role in stimulus-reinforcement learning. In the amygdala, information about the conditioned stimulus and unconditioned stimulus converge (Blair, 2017). Reduced amygdala responsiveness to the distress of other individuals has been shown in children with conduct problems and callous-unemotional traits (Marsh et al., 2008). If the amygdala is important for stimulus-reinforcement learning (Blair, 2017) and stimulus-reinforcement learning is a core requirement for appropriate moral judgments (Blair, 2007), then the amygdala should be involved when individuals are making moral judgments (Blair, 2017). It has been shown that in addition to the amygdala other regions are activated during moral judgement tasks, including the orbitofrontal cortex, insula, anterior cingulate cortex, precuneus and posterior cingulate cortex (Boccia et al., 2017). In agreement, adolescents with conduct problems and psychopathic traits showed reduced amygdala activity and reduced amygdala-orbitofrontal cortex functional connectivity when making judgements about legal/illegal actions (Marsh et al., 2011). The authors suggest that psychopathic traits may affect adolescents’ ability to attach the appropriate affective valence to actions of varying moral permissibility, and from using information about valence to guide their decisions (Marsh et al., 2011). In addition, in a study with incarcerated male adolescents fMRI was used while they viewed unpleasant pictures that did or did not depict moral transgressions and rated each on moral violation severity. While viewing unpleasant pictures with and without moral transgressions, correlations between hemodynamic responses in the amygdala and severity of moral violations ratings were positive in low callous-unemotional adolescents and negative in high callous-unemotional adolescents (Harenski et al., 2014).

In summary, a cognitive neuroscience perspective on moral thinking adds to the social-cognitive approach by drawing attention to care-based morality. Care-based morality refers to those forms of moral reasoning that concern actions that harm others (Blair, 2007). Deviances in perception of harm and moral transgressions have been found in adolescents with conduct problems and psychopathic traits.

## Including Goals, Outcome Expectations, and Normative Beliefs About Aggression in CBT Sessions with Parents and Child

The social problem-solving based CBT model includes nine psychological steps or skills (Matthys & Schutter, 2022, 2023) (Table 1). A distinction can be made between the first four steps which D’Zurilla and Goldfried (1971) would consider to belong to ‘general orientation’ (see Matthys & Schutter, 2022, 2023) and which we here call ‘preparatory steps’ on one hand, and the five core problem-solving steps on the other hand. Indeed, before actually starting to solve social problems (steps 5–9) children must learn which social situations are problematic for them (step 1), recognize problem situations by paying attention to facial expressions (step 2), becoming aware of one’s own emotions and regulate these emotions (step 3), and inhibit the tendency to respond on the first impulse as well as to concentrate on the problem (step 4) (Matthys & Schutter, 2022, 2023). The five core problem-solving skills are: interpretation (step 5), clarification of goals (step 6), generation of solutions (step 7), evaluation of solutions (step 8), and decision-making (step 9). Working on these nine psychological abilities only during CBT sessions may not be sufficient and needs to be continued in everyday life (“in vivo practice”; Kazdin et al., 1989). Parents, therefore, must be involved in the delivery of CBT of their child in view of supporting the learning processes involved in growth of social problem-solving abilities.

CBT can be offered individually or in a group; group format is engaging for children and offers opportunities for modeling. With regard to number of sessions, we suggest that the therapist works with the individual child or a small group of children on the nine skills in a minimum of 14 sessions: one session is spent on each of the four preparatory steps (1–4) and two sessions are spent on each of the five core social problem-solving steps (5–9) (Matthys & Schutter, 2022, 2023; Table 1). Before starting CBT with the child, the therapist explains the model in a psychoeducational session with both the child and the parents. In case of group format, the psychoeducational session can be offered to all participating children and their parents.

Next, we will focus on how parents can be involved in the delivery of CBT of their child after the psychoeducational session. Rather than working on each of nine psychological functions mentioned above in separate sessions with parents, we suggest parents and children work together in CBT sessions on three schemas (goals, outcome expectations, and normative beliefs about aggression) which simplify the cognitive tasks involved in the five core social problem-solving skills (i.e., interpretation, clarification of goals, generation of solutions, evaluation of solutions, decision-making) (Fig. 1). Children participate in individual sessions with their parents in view of tailoring CBT to the individual characteristics of the child’s schemas and social problem-solving skills. With regard to planning (Fig. 1), the session on Goals is scheduled after the child session on Generation of solutions. The session on Normative Beliefs is planned after the child session on Evaluation of solutions. Finally, the session on Outcome expectations is planned after the child session on Decision-making.

With regard to *goals*, social-cognitive research suggests that aggressive and delinquent children construct goals that are characterized as hostile, punishing, revengeful, dominant, conflict escalating, and focused on status and power. In contrast, when compared to well-adjusted children aggressive and delinquent children’s goals are characterized as less affiliative, relationship building and enhancing, conflict avoidant and aimed at closeness. As a result, aggressive and delinquent children will select goals that are characterized as hostile, punishing, revengeful, dominant, conflict escalating, and focused on status and power, and generate aggressive and antisocial solutions rather than prosocial solutions to social problems.

Discussing goals in CBT sessions with parents can help children in coming up with appropriate goals and generating adequate solutions to social problems (Fig. 1). When introducing the topic ‘goals’ the therapist uses the term ‘we’ (“When we have a problem …”) in order to avoid a defensive reaction from the child; this is critical especially for adolescents who may feel more defensive than children. After all, parents also can benefit from using the conceptual framework. “When we have a problem, it may be helpful to ask what is our goal: What is the goal we are aiming at? What do we want to accomplish? If we know our goals, we can better think about what to do.” Socratic questioning can be used to broaden children’s thinking and to access new knowledge. Socratic questions ignite children’s curiosity and wonder. Instead of offering interpretations to children, Socratic questions aim to help children arrive at their own interpretations (McLachlan et al., 2016). In view of making Socratic questions accessible for children, it is recommended to ask short, simple open-ended questions and avoiding why questions (Friedberg & McClure, 2015; McLachlan et al., 2016). Socratic questioning is used in order to find out characteristic goals of the child (e.g., What would you want to achieve in that situation?), encourage reflection on these goals (What would that lead to?), and explore alternative goals that had previously been outside the child’s attention (Maybe there are other things you could accomplish?). Coming up with alternative goals can ultimately result in the generation of appropriate responses.

With regard to *normative beliefs about aggression*, social-cognitive research suggests that the belief that aggression is legitimate or appropriate may lead to aggressive behavior through hostile interpretation, generation of aggressive solutions, positive evaluation of aggressive solutions, and selection of aggressive solutions among several solutions. A cognitive neuroscience perspective on morality adds to the social-cognitive approach by drawing attention to care-based morality. Care-based morality refers to those forms of moral reasoning that concern actions that harm others. Deviances in perception of harm and moral transgressions have been found in adolescents with conduct problems and psychopathic traits.

Discussing normative beliefs in CBT sessions with parents can help children interpret problem situations, generate solutions, evaluate solutions, and select solutions (Fig. 1). The therapist introduces these topics as follows: “Some children think it is okay to scream at a child when this child has said something bad first. These children think it is okay to scream at the child or to threaten to hit the child because they are convinced that’s the way people treat each other. When children think that only harsh or tough solutions work, they will decide to use these kind of solutions.” Socratic questioning can be used in order to find out characteristic normative beliefs of the child and encourage reflection on these beliefs (Friedberg & McClure, 2015; McLachlan et al., 2016). For example, when another child has done something bad to this child, and the child is convinced that the other child did that on purpose because that is the way people treat each other, the therapist can ask: “Are you sure about that?” “How can you be so sure?” “Is another explanation possible?” “Could it have happened by accident?” Thus, the therapist serves as a model for parents to discuss this schema with their child. Likewise, with regard to care-based morality, the therapist gives examples of moral transgressions and asks questions about the child’s perception of harm.

With regard to *outcome expectations*, social-cognitive research suggests that children with conduct problems expect that aggression will produce tangible rewards, will reduce aversive treatment by others, increases self-esteem and helps avoid a negative image (or maintains status among peers). Callous-unemotional traits are associated with decreased concern that aggressive acts will result in victim suffering. In addition, neurocognitive research shows deviances in the representation of the values (both rewarding and punishing) of the consequences of an action. As a result, children’s aggressive and antisocial behavior may be less planned than it may first seem. In addition, the decreased responsiveness to the consequences to the victim and difficulties in stopping or altering aggressive behavior can increase the damage aggressive and antisocial behavior cause.

Discussing outcome expectations in CBT sessions with parents can help children in generating and evaluating solutions, and making an appropriate decision (Fig. 1). The CBT therapist introduces these topics as follows: “After you have come up with solutions to the problem you can ask questions such as: ‘What do I think will happen if I do or say that? Will that help solve the problem? What is the direct effect for myself and for the other? And what is the effect in a week or a month? Do I not harm the other person with this solution? Is it correct to do that?’ It is indeed important to think about the consequences of your behavior, in the short and long term.” Socratic questioning can be used in order to find out characteristic outcome expectations of the child and encourage reflection on these outcome expectations (Friedberg & McClure, 2015; McLachlan et al., 2016). For example, the therapist can open a discussion as follows: “There are tough solutions to a social problem. What are the consequences of tough solutions?” The therapist then asks the question: “There are also kind solutions to social problems. Let us talk about kind solutions and see if they are likely to work or not.”

When clearly deviant schemas have been identified, more sessions on the schema are needed. With a view to a further process of adapting the schema in the child, therapists function as a model for parents to ask their child questions about the relevant schema. The therapist notes questions that parents can ask children about their schemas with a view of achieving changes in their schemas. Attention also needs to be paid to possible deviant schemas among parents as these can maintain children’s schemas.

## Discussion

Children with conduct problems derive increasing benefit from direct participation in CBT with increasing age, wherein social problem-solving is targeted (Fairchild et al., 2019). According to British guidelines, group social and cognitive problem-solving programs for the treatment of conduct problems should be based on a cognitive-behavioral problem-solving model (National Institute for Health and Clinical Practice (NICE), 2013, 2017; Pilling et al., 2013). Based on a model of CBT (Matthys & Schutter, 2022, 2023) and evidence for the effect of three maladaptive schemas on five core social problem-solving skills, we here offer suggestions how therapists can assist children and parents in changing children’s schemas. This approach does not exclude, however, the need to also work on parenting skills (Beelman et al., 2023) or, in case of adolescents, on problems in multiple systems (family, peers, school, community) (Henggeler et al., 2009; Van der Stouwe et al., 2014) when in the clinical assessment these have been found to play a role in maintaining conduct problems (Matthys & Powell, 2018).

The schemas to be discussed in therapy sessions with children and parents all have a moral character. Morality is about values such as the well-being of others, the rights of others, honesty and justice. These moral values become visible in normative behavior such as helping others. Moral values (e.g., the well-being of others) underlie goals (e.g., relationship building versus revengeful goals), as well as outcome expectations (e.g., concern about the other’s distress versus not worrying about harming others) and normative beliefs (e.g., kind solutions versus aggression solutions work). In discussions with the child about moral issues there is a risk that a punitive approach by parents prevails. When parents talk to their children in punitive ways this diminishes children’s other orientation by leading them to focus on their own negative affect (Recchia & Wainryb, 2023). As a result, children are less likely to explore the psychological facets of their experiences that account for their actions (Recchia & Wainryb, 2023), such as their goals, normative beliefs, and outcome expectations. Adolescents may feel even more defensive than children when parents participate in the sessions. Although therapists create a climate in which both children or adolescents and parents are encouraged to talk about their experiences and related thoughts and feelings, separate meetings of the adolescent and the therapist may be needed.

The three schemas discussed here are based on social-cognitive research and cognitive neuroscience research. These two approaches complement each other. For example, social-cognitive research provides extensive information about goals and related starting points for interventions. Aggressive children’s goals are characterized as hostile, punishing, revengeful, dominant, conflict escalating, and focused on status and power. In interventions these need to be changed to goals that are affiliative, relationship building and enhancing, conflict avoidant and aimed at closeness. If children with conduct problems learn to use these goals in real life interactions this may help them solve social problems more appropriately. On the other hand, cognitive neuroscience research conceptualizes goals in terms of outcomes to be expected (Blair, 2022). Indeed, aggression, in particular instrumental aggression and reactive aggression with an instrumental/proactive component, is a behavior motivated by the desire to achieve a particular goal (Blair, 2022). Cognitive neuroscience research investigates aggressive children’s problems with the representation of the values of the consequences of an action. As a result of difficulties in the representation of the value (rewarding/positive, punishing/negative) of the consequences of actions, aggressive and antisocial behavior may be less instrumental, planned or proactive than it may first seem.

To the best of our knowledge, studies on goals, outcome expectations and normative beliefs in parents and siblings of children with conduct problems are currently lacking. One may expect that in some families the child with conduct problems is not the only one with deviant goals, outcome expectations and normative beliefs. If so, more time will be needed to reach consensus, if at all, among the family members on these schemas.

There has been much discussion about the role of callous-unemotional traits in treatment responsiveness of children with conduct problems (Fleming, 2023; Frick, 2023). A recent meta-analysis, however, showed similar treatment-related reductions in children with conduct problems with and without callous-unemotional traits, although children with callous-unemotional traits started and ended treatment with more behavior problems (Perlstein et al., 2023). The authors conclude that personalized adjunctive treatment modules are warranted to bring about greater improvement in children with callous-unemotional traits (Perlstein et al., 2023).

## Conclusion

In view of supporting the child’s learning processes regarding social problem-solving we suggest that parents and the child work together with the therapist on three schemas (goals, outcome expectations, and normative beliefs about aggression) which simplify the cognitive tasks involved in the five core social problem-solving skills (interpretation, clarification of goals, generation of solutions, evaluation of solutions, decision-making). These sessions are tailored to the individual child’s characteristic schemas and associated social problem-solving skills. The therapist uses Socratic questioning in order to find out characteristic schemas of the child, encourage reflection on these schemas, and explore alternative schemas that had previously been outside the child’s attention. The therapist functions as a model for parents to ask their child questions about the relevant schemas with a view of achieving changes in the schemas.

## Data Availability

Data sharing not applicable to this article as no datasets were generated or analysed during the review.
